# Sperm Cyst “Looping”: A Developmental Novelty Enabling Extreme Male Ornament Evolution

**DOI:** 10.3390/cells10102762

**Published:** 2021-10-15

**Authors:** Zeeshan A. Syed, Romano Dallai, Negar Nasirzadeh, Julie A. Brill, Patrick M. O’Grady, Siyuan Cong, Ethan M. Leef, Sarah Rice, Amaar Asif, Stephanie Nguyen, Matthew M. Hansen, Steve Dorus, Scott Pitnick

**Affiliations:** 1 Center for Reproductive Evolution, Department of Biology, Syracuse University, Syracuse, NY 13244, USA; sicong@syr.edu (S.C.); emleef@syr.edu (E.M.L.); srice10@binghamton.edu (S.R.); amasif@syr.edu (A.A.); snguye01@syr.edu (S.N.); hansenmatt@outlook.com (M.M.H.); sdorus@syr.edu (S.D.); 2 Department of Life Sciences, University of Siena, via Aldo Moro 2, 53100 Siena, Italy; romano.dallai@unisi.it; 3 Cell Biology Program, The Hospital for Sick Children, Toronto, ON M5G 0A4, Canada; negar.nasirzadeh@mail.utoronto.ca (N.N.); julie.brill@sickkids.ca (J.A.B.); 4 Department of Molecular Genetics, University of Toronto, Toronto, ON M5S 1A8, Canada; 5 Department of Entomology, Cornell University, Ithaca, NY 14853, USA; pmo43@cornell.edu

**Keywords:** postcopulatory sexual selection, female reproductive tract, life-history, energetic constraint, spermatogenesis, trait diversification, heterochrony, willistoni, saltans

## Abstract

Postcopulatory sexual selection is credited as a principal force behind the rapid evolution of reproductive characters, often generating a pattern of correlated evolution between interacting, sex-specific traits. Because the female reproductive tract is the selective environment for sperm, one taxonomically widespread example of this pattern is the co-diversification of sperm length and female sperm-storage organ dimension. In *Drosophila*, having testes that are longer than the sperm they manufacture was believed to be a universal physiological constraint. Further, the energetic and time costs of developing long testes have been credited with underlying the steep evolutionary allometry of sperm length and constraining sperm length evolution in *Drosophila*. Here, we report on the discovery of a novel spermatogenic mechanism—sperm cyst looping—that enables males to produce relatively long sperm in short testis. This phenomenon (restricted to members of the saltans and willistoni species groups) begins early during spermatogenesis and is potentially attributable to heterochronic evolution, resulting in growth asynchrony between spermatid tails and the surrounding spermatid and somatic cyst cell membranes. By removing the allometric constraint on sperm length, this evolutionary innovation appears to have enabled males to evolve extremely long sperm for their body mass while evading delays in reproductive maturation time. On the other hand, sperm cyst looping was found to exact a cost by requiring greater total energetic investment in testes and a pronounced reduction in male lifespan. We speculate on the ecological selection pressures underlying the evolutionary origin and maintenance of this unique adaptation.

## 1. Introduction

Landmark theoretical contributions by Geoff Parker, starting in the 1970s and continuing to this day, have provided the perfect complement to Darwin’s [1,2] treatment of premating sexual selection. In particular, postcopulatory sexual selection theory, which includes the combinatorial selective effects of sperm competition [3] and cryptic female choice [4], and sexual conflict theory [5] have proven critical for understanding the origin and evolutionary maintenance of anisogamy [6,7,8], as well as the extraordinary diversification of gametes and other reproductive characters [9,10,11,12,13,14].

Although our understanding of the precise role of sexual selection in driving the evolution of sperm form and function is still incomplete [15], the selective role of the female reproductive tract, in the case of internally fertilizing species, is clear. A recent comparative analysis of 3233 species across 21 animal phyla, from sponges to chordates, revealed that sperm length has diverged more rapidly and extensively following independent origins of internal fertilization [16]. Moreover, morphological co-diversification of sperm length and some critical dimension(s) of the female reproductive tract (e.g., sperm-storage organ size or duct length) is one of the most taxonomically widespread patterns in the field of comparative reproductive biology [12,17].

The experimental system for which the evolution of sperm form has been most intensively investigated is the fruit fly, *Drosophila melanogaster*, and its relatives. Comparative analyses, quantitative genetics, experimental evolution, and functional analyses have all provided complimentary demonstrations that the length of the female’s primary sperm-storage organ, the seminal receptacle (SR), generates selection on sperm length, thus contributing to the co-diversification of these functionally interacting, sex-specific traits [18,19,20,21,22,23,24]. This selective process presumably underlies the multiple independent evolutionary origins of giant sperm across the *Drosophila* phylogeny [20,25]. 

SR length is the most rigorously demonstrated mechanism of cryptic female choice in *Drosophila*. Sperm length is the postcopulatory ornament/armament that is the target of this female preference. The relative length of the SR determines the extent of female discrimination for sperm length [18,21,22,24,26]. Sexual selection theory predicts that the evolution of exaggerated preferences, armaments, and ornaments can be constrained by balancing natural selection that imposes resource limitations and other costs on the development and maintenance of such traits [27,28,29]. Previous studies have demonstrated substantive costs of longer SRs and sperm. Growth of the SR is significantly condition-dependent [30], and the experimental evolution of exaggerated SR length results in a significant correlated delay in egg-to-adult development time and decreased longevity of mated (but not virgin) female *D. melanogaster* [31]. Similarly, exaggerated sperm length across the *Drosophila* species is positively correlated with increased energetic investment in testes, despite reduced sperm production, and with delayed male post-eclosion maturation time [21,32,33,34,35,36,37]. Previous investigations have interpreted the strong, positive evolutionary allometry of sperm length in *Drosophila* to be a consequence of these energetic costs, with males of larger-bodied species better able to “afford” to respond to selection for increased sperm length when it arises [20,21,25,35].

In contrast to this knowledge about the pattern and process of sperm and SR length evolution, there is nearly a complete absence of information regarding the developmental mechanisms underlying interspecific variation in sperm and female sperm-storage organs in *Drosophila* or any other taxa [38]. Few studies have identified the genetic basis of variation in sperm form [39,40], and no researchers have capitalized on the extraordinary variation in sperm form [9] by treating spermatogenesis per se as a model system for evolutionary developmental (evo-devo) analysis of cell morphogenesis. Here, we report on the discovery of a novel spermatogenic mechanism—sperm cyst “looping” (henceforth “cyst looping”)—that is unique to the monophyletic lineage of *Drosophila* comprising the *willistoni* and *saltans* species groups, and which appears to enable males of these species to produce relatively long sperm in short testes. We further explore the adaptive value of cyst looping and its relationship to sperm length evolution by quantifying and comparing patterns of divergence in numerous other reproductive traits (i.e., SR length, testis length, relative testis mass, number of sperm transferred per copulation, and female remating interval) and life-history traits (i.e., body mass, male age at first reproduction and sex-specific longevity) within a phylogenetic analytical framework. Trait relationships were examined across three discrete lineages: (1) the *willistoni* species group (seven species investigated), for which all species exhibit “complete” cyst looping, (2) the *saltans* species group (five species investigated), for which all species exhibit “partial” cyst looping and (3) an assortment of outgroup species (16 species investigated), which exhibit no cyst looping.

## 2. Materials and Methods

### 2.1. Experimental Organisms and Maintenance

Cultures of all 28 species investigated were acquired from the National *Drosophila* Species Stock Center (NDSSC) at Cornell University, New York, NY, USA (stock numbers are provided in SI). All species were reared under standardized conditions of overlapping generations and an approximate 1:1 sex ratio in glass half-pint bottles containing approximately 30 mL of culture medium supplemented with a sprinkle of live yeast granules. All stocks were maintained at 22.5 ± 0.5 °C at an approximately 12:12 photoperiodic cycle, with adult densities conducive to moderate larval density. All species were reared on “NDSSC cornmeal” culture medium (for the recipe, see http://blogs.cornell.edu/drosophila/recipes/, accessed on 1 July 2018). Unless mentioned otherwise, experimental flies were collected as virgins within 12 h of eclosion and stored in 8-dram plastic shell vials with medium and live yeast, with 10–20 other same-sex individuals (higher density with smaller-bodied species) until reproductively mature. For species with longer maturation times, flies were transferred to fresh vials twice a week. Flies were anesthetized before collection using CO_2_ and before dissection using ether.

### 2.2. Ultrastructure of Sperm Cysts and the Seminal Receptacle

Following respective dissections of *D. willistoni* and *D. saltans’* testes and the lower reproductive tracts of females into 0.1 M phosphate buffer solution (PBS; pH 7.2) containing 3% sucrose, tissues were fixed in 3% glutaraldehyde in PBS at 4 °C. After washing in PBS, the material was post-fixed in 1% osmium tetroxide in PBS for 1–2 h, carefully rinsed, dehydrated in a graded series of ethanol (50–100%), and then transferred into a mixture of propylene oxide and Epon-Araldite (50/50) and cured at 4 °C for a day. On the following day, the material was transferred into a pure mixture Epon-Araldite resin and embedded in small silicon molds to form blocks after 48 h polymerization in oven at 60 °C. Semithin sections of testes and SRs, obtained with an ultramicrotome Reichert Ultracut, were stained with 0.1% toluidine blue and photographed at a Leica DMRB light microscope equipped with a Zeiss AxioCam Digital Camera. Ultrathin sections were stained with uranyl acetate and lead citrate and observed at a CM10 Philips transmission electron microscope operating at an electron accelerating voltage of 80 kV.

### 2.3. Immunofluorescence Investigation of Sperm Cyst Development

Immunofluorescence was performed as described by Hime et al. (1996) [41]. Briefly, testes of newly eclosed (1–2 day old) male flies were dissected in testis isolation buffer [42] and squashed with coverslips on Polysine Adhesion Slides (ThermoFisher Scientific, Portsmounth, NH, USA, Cat#: P4981-001). Samples were frozen in liquid nitrogen, coverslips were removed, and samples were placed in chilled 95% ethanol for a minimum of 10 min. Samples were then fixed using 4% paraformaldehyde (Electron Microscopy Sciences, Hatfield, PA, USA, Cat#: 15710) for 7 min and briefly washed with PBT (PBS and 0.1% Triton X-100), followed by two 15 min washes in PBT DOC (PBS, 0.1 Triton X-100, and 0.3% sodium deoxycholate). Following two 5 min washes with PBT, samples were blocked for 30 min in PBT BSA (PBT and 5% bovine serum albumen) and incubated overnight at 4 °C with primary antibodies. The next day, samples were washed with PBT, incubated at room temperature for 1 h with secondary antibodies, then washed twice in PBT for 15 min, and mounted using ProLong^TM^ Glass Antifade Mountant with NucBlue^TM^ (Invitrogen, Eugene, OR, USA, Cat# P36985). Antibodies used were as follows: 1:1000 mouse anti-acetylated tubulin (Sigma, St. Louis, MO, USA, Cat#: T7451); 1:1000 goat anti-mouse secondary antibody conjugated to Alexa Fluor 488 (Invitrogen, Eugene, OR, USA, Cat#: A11001). Preparations were examined on an inverted Leica DMi8 epifluorescence microscope equipped with LED3 light source, Leica K5 camera and THUNDER Imaging System. Images were acquired and adjusted for brightness and contrast using LAS-X software (Version 3.7.4.23463, Leica Microsystems Ltd., Wetzlar, Germany) and ImageJ (Fiji Version 2.1.0/1.53c) [43]. The figure was created using Adobe Illustrator (Version 25.4.1 (2021), Adobe Inc., San Jose, CA, USA).

### 2.4. Phylogeny

Ingroup taxa were selected to show a representation of major lineages (*melanogaster*, *saltans*, *willistoni*) within the subgenus *Sophophora* [44]. Eight members of the subgenus *Drosophila*, representing the *virilis* and *immigrans* species groups, were used as outgroup taxa. Sequences from 23 nuclear genes (*18S*, *28S*, *aatshis*, *Adh*, *Amd*, *amyrel*, *aralar1*, *atub84b*, *bap60*, *cg7843*, *cora*, *Ddc*, *esc*, *fkh*, *Gpdh*, *ksr*, *ost48*, *PGI*, *hpo*, *snf*, *sod*, *TPI*, *Xdh*) and partially complete mt genomes were obtained from NCBI and aligned using MAFFT (Version 7) [45]. The total alignment length was 489,234 base pairs. Phylogenetic trees were constructed using RAxML (version 8.2.7) [46] in Geneious Prime (Version 11.1.4; Biomatters, Inc., San Diego, CA, USA). The initial phylogeny was constructed using 32 species. However, we had comparative data for 28 species; four species for which we did not have data were thus pruned from the tree for analyses. Nonparametric bootstrap support values for all nodes are presented for the larger tree in the Supplementary Materials (Figure S1).

We reconstructed a time calibrated phylogeny (ultrametric tree) of the sampled species (ultrametric) using penalized likelihood as implemented in the function ‘chronos’ within the R package ‘ape’ [47,48,49]. We used a secondary node constraint approach by supplying, for the input, previously estimated minimum and maximum ages for the following nodes: the root of the phylogeny (Drosophilidae, 22.7–60 mya), the node ancestral to the *willistoni-saltans* lineage and the *melanogaster* lineage (22.2–46.8 mya), and the node ancestral to the subgenera *Sophophora* and *Drosophila* (34–50 mya), which were obtained from existing literature [50,51]. The smoothing factor (λ) was set to zero and the model of substitution rate variation among branches was set as “relaxed”, based on the ϕ Information Criterion (PHIIC) as described in [49].

### 2.5. Data Acquisition

All morphometric measurements were obtained using the software Fiji [43] with photomicrographs (differential interference contrast [DIC], darkfield or fluorescent) captured using an Olympus DP-71 or an Infinity (Teledyne Lumenera) camera mounted on either an Olympus SZX12 stereomicroscope or a BX60 compound microscope. Data for specific traits were collected as follows.

#### 2.5.1. Sperm Cyst Looping (*n* = 5 Males per Species)

**Figure 1:**
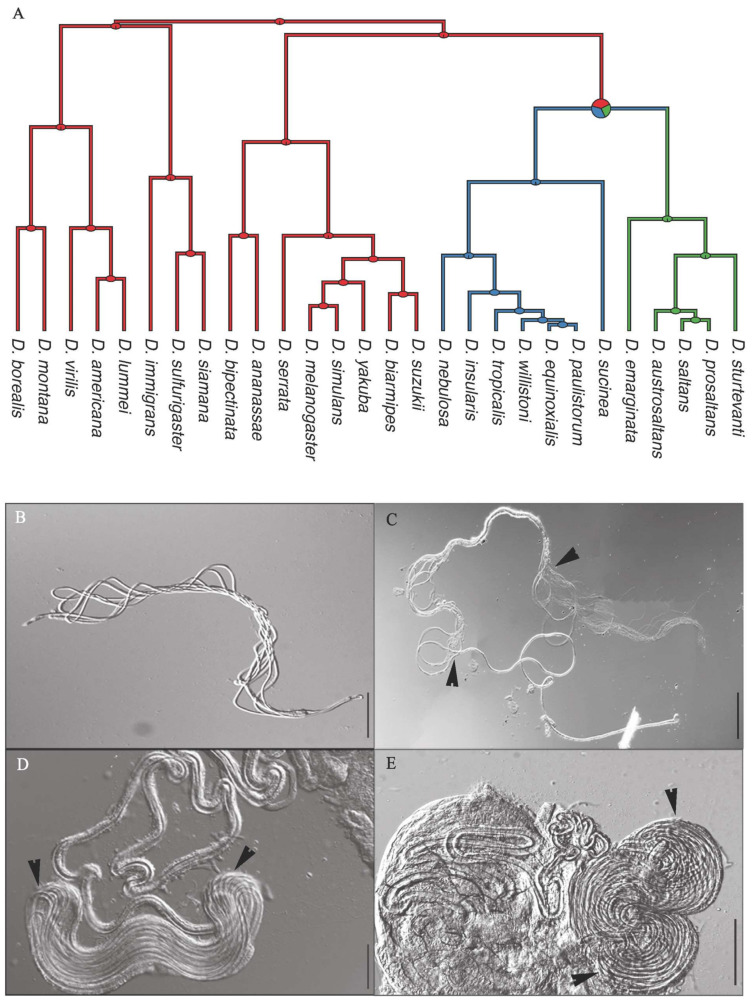
Phylogeny of the 28 examined *Drosophila* species showing the ancestral state reconstruction of sperm cyst looping (**A**; blue = complete cyst looping, green = partial cyst looping, pink = cyst looping absent) and DIC stereomicrographs showing representative images of sperm cysts (**B**,**C**) and female seminal receptacles (SRs; (**D**,**E**)). (**B**,**D**) *D. equinoxialis* (in the *willistoni* species group), which exhibits complete cyst looping. (**C**,**E**) *D. saltans*, which exhibits partial cyst looping. Arrows in **C** bracket the looped portion of the cyst. Arrows in **D,E** indicate the highly integrated loops of the distal portion of the SR (also see Figure 4). Scale bars = 100 µm.

We categorized the state of cyst looping for each species as either ‘complete,’ ‘partial’ or ‘absent.’ With complete cyst looping, the entire sperm cyst is looped back and forth, with all loops of approximately equal length (Figure 1B). With partial cyst looping, only a medial region of the cyst is looped, with a lengthy portion of both the anterior and posterior ends of the cyst extending beyond the looped region (Figure 1C). Species were designated as absent for cyst looping when no region of cysts were ever observed to form loops.

#### 2.5.2. Sperm Length, Loop Length, and the Number of Loops per Cyst (*n* = 3–5 Males per Species)

Values were estimated from mature syncytial cysts. The samples were prepared by largely following methods described by Pitnick and Markow [34]. Briefly, a few cysts from a testis of a sexually mature male were carefully dissected and isolated on a subbed (i.e., coated with gelatin) glass slide containing a drop of PBS. Slides were then dried for several hours at 70–75 °C prior to fixation using 3:1 methanol-acetic acid solution and staining with DAPI. Only the most mature cysts—those extending into the proximal end (adjacent to the seminal vesicle)—were measured. Loop length was determined by measuring the length between two opposite hairpin bends (Figure 1B,C). The number of loops was estimated by counting the number of hairpins in each cyst. In species where cyst looping is absent, total sperm lengths were previously reported for some species by Pitnick et al. [25]. For all other species, novel data are reported here. For species with relatively short sperm (i.e., <5 mm), sperm from seminal vesicles were gently freed into PBS, fixed, and stained as described by Pitnick et al. [25]. Darkfield images of individual sperm from seminal vesicles were captured at 100x or 200x depending on length and layout. For species with relatively long sperm (i.e., >5 mm), length was estimated by measuring mature sperm cysts as described above for species with cyst looping. Total sperm and cyst lengths were measured by tracing the axis using the segmented line tool of Fiji. Five sperms or cysts were measured per male with the mean for each male calculated using the four longest measures.

#### 2.5.3. Seminal Receptacle Length (*n* = 5 Females per Species)

Each reproductive tract was dissected intact from a sexually mature, inseminated female into PBS on a subbed microscopic slide. The lower reproductive tract was separated from the ovaries by severing the common oviduct. Fine forceps and pins were used to break tracheoles binding regions of the SR together (restricted to the proximate portion of the SR only for *saltans* and *willistoni* group species; Figure 1D,E), thus allowing the organ to extend away from the bursa for better visualization and measurement. A glass coverslip with clay feet was placed over the preparation and gently compressed to render the SR two-dimensional without over-compression and DIC images were captured (100x or 200x depending on length and layout; lengthy SRs required multiple images to capture the entire length). For species with exceptionally long SRs organized into tightly bound loops (see below), length measurements were obtained by measuring the average length of one loop and then multiplying by the number of loops.

#### 2.5.4. Testis Length (*n* = 3–5 Males per Species)

The reproductive tract was dissected into PBS on a microscopic slide. For species with lengthy testes, tracheoles binding together loops of each testis were severed to better spread the testes out on the slide for visualization. A glass coverslip with clay “feet” was used to make the tissue two-dimensional without over-compression (see above) and DIC images were captured (at 100x or 200x magnification) of at least one testis, following which its length was determined by tracing the central axis from the distal tip to the juncture of the testis and the seminal vesicle using the segmented line tool of Fiji.

#### 2.5.5. Thorax Length and Gonadosomatic Index (GSI) (*n* = 5 Males per Species)

Thorax length (anterior margin of the mesonotum to the posterior tip of the mesoscutellum, in lateral view) was measured using the ocular micrometer of a stereomicroscope at 63x (larger species) or 80x magnification (smaller species). Following measurement, both testes from each male were dissected in distilled water and then transferred to a small, pre-weighed square of aluminum foil; all remaining tissue was placed on another pre-weighed piece of foil. After drying, samples were weighed on a Cahn C-35 microbalance to the nearest 1.0 µg, and then foil weights were subtracted to determine tissue mass. GSI was calculated as dry testis mass (dry body mass + dry testis mass). Thorax length and body mass of females were measured the same way as males, except whole females were dried and weighed.

#### 2.5.6. Male Post-Eclosion Maturation Time

For each species, 70 first-instar larvae were placed in an eight-dram plastic vial containing 2 mL cornmeal molasses medium. Males were collected within 4 h of eclosion and held in vials provisioned with cornmeal molasses food supplemented with live yeast grains. Males were randomly selected for dissection in batches of 10 at 12-h intervals. Sexual maturity was defined by the presence of mature, individualized sperm in the seminal vesicles. Time to sexual maturation for a species was noted as the first time point when 8 out of 10 males were found to be sexually mature [25].

#### 2.5.7. Sex-Specific Lifespan (*n* = 80 per Sex per Species)

On the day of eclosion, 40 males and 40 females were placed into each of two replicate 850 mL plastic boxes provisioned with 30 mL of cornmeal media sprinkled with live yeast in a petri dish (diameter = 10 cm) attached to the bottom of the box using double-sided tape. The mouth of each box was covered with a nylon stocking to facilitate airflow and to enable the switching of food plates and the aspiration of dead flies without letting the live flies escape. Twice weekly, the food dish was replaced, and all dead flies were removed from each chamber. All fly carcasses were examined under a stereomicroscope to determine sex; for species lacking sexual dimorphism, carcasses were first rehydrated in PBS with 1% Triton-X 100 (Sigma Aldrich Inc. St. Louis, MO, USA) to facilitate visualization of external genitalia. Assays continued until every fly in a chamber was dead.

#### 2.5.8. Number of Sperm Transferred per Copulation (*n* = 5 per Species) and Female Remating Interval (*n* = 25 per Species)

All matings were conducted during the morning with groups of flies. Five virgin, reproductively mature males and females were combined in each vial with medium and observed for copulation. Once a pair was observed to begin mating, all other flies were gently transferred into a new vial by aspiration without disturbing the mating pair. As the assay progressed, unmated flies were combined to maintain a roughly 5:5 ratio. Start and end times for all copulations were recorded. The number of vials initially set up (10 minimum) differed among species depending on their respective mating propensity, but enough mating vials were set up for each species to obtain 50 matings. Immediately afterward, copulating males were discarded, and females were retained individually in their vials. Two or three females from the matings with the lowest copulation duration were dissected and checked for presence of sperm in the bursa and/or SR to eliminate any pseudocopulations (i.e., failure to transfer an ejaculate). The remaining mated females were randomly assigned to one of two phenotyping assays. First, to determine the number of sperm transferred per copulation, some females were flash-frozen in liquid nitrogen immediately after mating and stored at −20 °C for later thawing and dissection. For each female, sperm from the bursa (and from sperm-storage organs when necessary for species with lengthy copulation durations) were dissected into PBS on a subbed microscope slide. Samples were then dried, fixed, and stained with DAPI following methods described above. Number of sperm heads were counted using a fluorescent microscope at 400x, aided by an ocular reticule grid. Second, 25 mated females were used to assay the remating interval. These females were equally distributed among 5 vials and combined with 8 virgin males in each vial, then continuously observed for any matings. The first assay took place during the afternoon on the same day of the “virgin” mating (day 0.5) and lasted 3 h. When a remating was observed, all non-mating flies were gently moved to another vial. Those females not remating by the end of the observation period on each day were separated from the males under light CO_2_ anesthesia, then combined with fresh young virgin males on the next day. The assay was repeated each morning only for 5 h, on days 1, 2, 3, 4, 7, 10, 14, 21 and 28 following the “virgin” mating, or until 13/25 females remated. The day on which the 13th female remated was designated as the median remating interval (RI_50_) of the species. Any species where less than 13 females remated by the 28th day post remating was categorized as monandrous.

### 2.6. Statistical Analyses

All analyses were performed in R version 4.0.3 [52]. Ancestral state reconstruction was performed using the ‘ace’ (Ancestral Character Estimation) function in the R package ‘ape’ using three models (equal rates, symmetric and all-rates-different) on the ‘discrete’ character variable [47]. The results of the equal rates model were chosen as the best model based on likelihood ratios.

Phylogenetic multivariate regressions between traits were calculated using phylogenetic generalized linear models (PGLS), using the R package ‘caper’ [53]. The pgls function in caper simultaneously estimates a maximum likelihood estimate for phylogenetic inertia (Pagel’s λ). Values of λ closer to 0 indicate independent evolution whereas values closer to 1 indicate phylogenetic signal. The effect of looping was modeled as a fixed categorical predictor and the phylogenetic slopes comparing among groups of species with complete, partial, or no sperm looping were compared using F-tests (anova.pgls function in ‘caper’). When examining (1) allometric relationships between SR or sperm length and thorax length, and (2) the evolutionary relationship between sperm length and SR length, cyst looping was categorized as ‘present’ (combining the partial and complete looping observed in the *willistoni* and *saltans* group species) or ‘absent.’ Similar categorization was used when analyzing female longevity, since we were testing the predicted life-history cost of the highly compact, looped SRs, which did not differ in appearance between the *willistoni* and *saltans* species groups (Figure 1D,E). When testing predictions about the life-history consequences of cyst looping for males, species were categorized as either ‘complete looping,’ ‘partial looping,’ or ‘looping absent’.

## 3. Results

Consistent with previous studies, our phylogenetic tree recovered the *willistoni* and *saltans* groups as monophyletic, forming a clade as sister groups with strong support values [44,54]. However, some subgroups within the groups had relatively lower support values. The topology of the outgroup species was also consistent with previous studies (Figure S1) [44,50,54,55].

**Figure 2:**
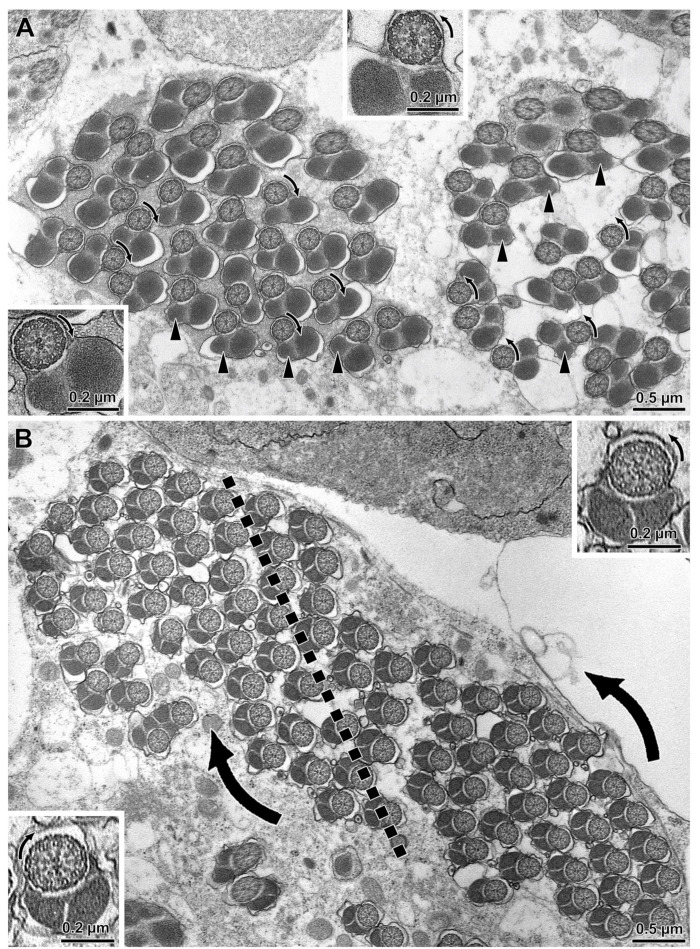
Cross section through two adjacent loops of the same cyst of (**A**) *D. willistoni* and (**B**) *D. saltans* illustrating the bidirectional orientation of axonemes between loops. Note the position of the smaller mitochondrial derivative (arrowheads) differs between loops in being on the left or on the right side. In the loop to the left of both images, the dynein arms of the flagellar axonemes are oriented clockwise, whereas in the loop to the right the dynein arms are oriented anti-clockwise (arrows). The former (clockwise) are flagella seen from the tail end towards the nuclear region, whereas the latter ones (anti-clockwise) are flagella seen from the nucleus towards the tail end. Insets are higher magnifications of the flagellar axonemes with different dynein orientation.

Using light microscopy to examine cysts dissected intact from testes, complete looping was observed in all seven assessed members of the *willistoni* species group, whereas partial looping was observed in all five assessed members of the *saltans* species group. Neither form of looping was observed in any of the outgroup species (Figure 1; Table 1). Transmission electron microscopy of the testes of *D. willistoni* and *D. saltans* confirmed the occurrence of cyst looping in situ for both species (Figure 2). Phylogenetic reconstruction supports the conclusion that some form of cyst looping originated in a node immediately ancestral to the division between the *saltans* and *willistoni* clades, with complete looping either subsequently gained by the *willistoni* species group or lost by the *saltans* species group. There is a small probability that members of the discrete ancestors of the *saltans* and *willistoni* groups evolved partial and complete looping, respectively, independent of one another (Figure 1A).

**Figure 3:**
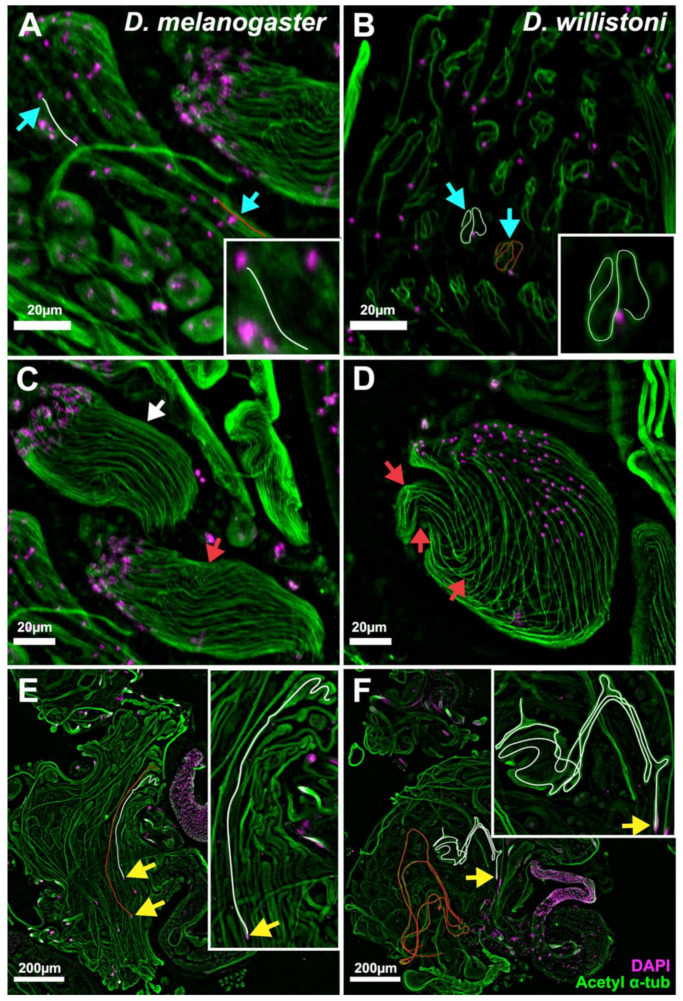
Epifluorescence micrographs of spermatid cysts from *D. melanogaster* (**A**,**C**,**E**) and *D. willistoni* (**B**,**D**,**F**) stained for acetylated α-tubulin (sperm tail, green) and DAPI (DNA, magenta). (**A**,**B**) Early stage of sperm tail elongation in a syncytial cyst of haploid spermatids. (**A**) *D. melanogaster* spermatid tails are straight (cyan arrows and trace lines). Both the cells and the sperm tails are elongated. (**B**) *D. willistoni* sperm tails are looped (cyan arrows and trace lines). The cells are still round, whereas the sperm tails have already elongated. (**C**,**D**) Slightly later stage of sperm tail elongation, showing groups of 64 spermatids encased in somatic cyst cells (cyst cells not shown). (**C**) *D. melanogaster* sperm tails are mostly straight (white arrow) with occasional kinks along their length (red arrow). (**D**) *D. willistoni* sperm tails are starting to bend and have multiple kinks along their length (red arrows). (**E**,**F**) Partially or fully elongated spermatid cysts. (**E**) *D. melanogaster* cysts are straight (trace lines). Note that the trace lines both start from a group of 64 sperm heads (yellow arrows) and extend along the entire length of each cyst. (**F**) *D. willistoni* cysts are looped multiple times (trace lines). Note that the white trace line covers the entire length of the cyst, starting at the 64 sperm heads (yellow arrows), but the red trace line covers only a portion of the cyst. For better visualization, images shown in (**A**,**C**) are maximum projections of z-stacks, whereas (**B**,**D**) are extended depth of field images processed using LAS-X software. (**E**,**F**) are single plane images. Insets are magnified 2.5 times compared to their corresponding images. Scale bars: 20 µm (**A**–**D**), 200 µm (**E**,**F**).

The propensity of *D. willistoni* sperm tails to form loops was evident from the earliest stages of elongation. At early stages (Figure 3A,B), *D. melanogaster* sperm tails were fully extended (Figure 3A, cyan arrows, traces), whereas *D. willistoni* sperm tails were looped (Figure 3B, cyan arrows, traces). The same trend was also observed at slightly later stages (Figure 3C,D); *D. melanogaster* sperm tails were mostly straight (white arrow), with only occasional kinks (Figure 3C, red arrow), whereas *D. willistoni* sperm tails, while more elongated than at earlier stages (Figure 3B), exhibited bending and looping (Figure 3D, red arrows). A comparison of later-stage elongated cysts of the two species (Figure 3E,F) revealed an absence of looping in *D. melanogaster* (Figure 3E, traces) and extensive looping in *D. willistoni* (Figure 3F, traces). We note that the squashing technique used to generate the samples can cause distortion in the morphology of elongated spermatid cysts. However, the presence or absence of loops and the difference in the degree of looping were easily distinguishable between the two species. Thus, in contrast to *D. melanogaster*, which consistently exhibits straight sperm tails, looping of *D. willistoni* sperm tails begins at the earliest stages and progresses throughout spermatid elongation.

**Figure 4:**
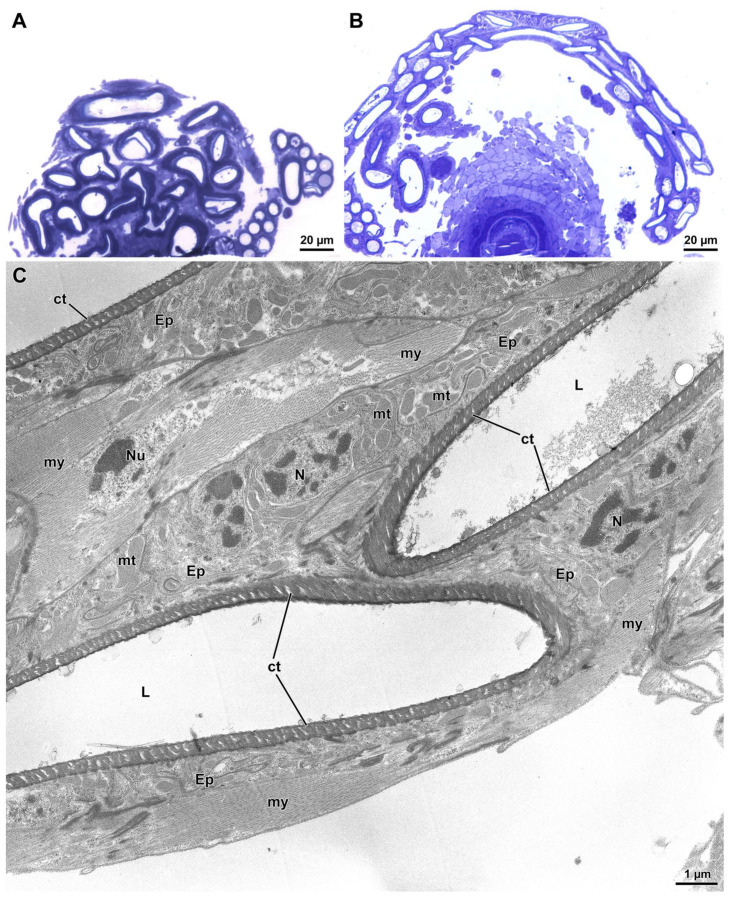
The seminal receptacle (SR) of *D. willistoni.* (**A**) Semithin cross section of the SR at its insertion on the ventral region of the genital uterine wall. Note that the several cross-sectioned tubular loops are interconnected by a tissue complex. (**B**) Semithin section through a more distal region showing the integration of loops within a tissue complex. (**C**) Ultrathin section of the distal SR revealing that tubular lumens (L) are formed by an irregular thin epithelial layer (Ep) lined by a cuticle (ct). The cytoplasm contains nuclei (N), mitochondria (mt), and scanty dense inclusions. Beneath the epithelial layer, polymorphic muscle cells are visible with their nuclei (Nu) and the myofibrils (my).

The SRs of the *willistoni* and *saltans* group species were similar to one another in terms of structure and organization, but different from those of all previously examined *Drosophila* species [20,56]. Interestingly, they also exhibit a tight looping structure, with adjacent loops integrated within a common tissue (Figure 1D,E and Figure 4). By contrast, all members of other *Drosophila* species groups have SRs for which the entire length of the tubule is free in hemolymph rather than embedded in tissue, and any physical association between different regions of the SR is accomplished exclusively by connecting tracheoles. The proximal region of the SR of *D. willistoni* forms a tight complex of loops, each 6.8 µm wide and 4.0 µm thick, over the whole anterior region of the ventral genital uterine wall (Figure 4A). Transmission electron microscopy reveals that, more distally, the multiple tubular loops are tightly connected by surrounding tissues (Figure 4B) with a thin layer of irregular epithelial cells (1.0–2.1 µm thick) surrounding tubule cross sections (2.0–2.5 µm diameter) that are lined by a thin cuticle (0.37 µm high). Beneath the epithelial cells, irregular muscle cells are visible, extending to contact the epithelia of the adjacent tubular loops (Figure 4C). This compact tissue complex accompanies the SR for the entirety of its length, being especially pronounced in the distal looped portion (Figure 1D,E).

**Figure 5:**
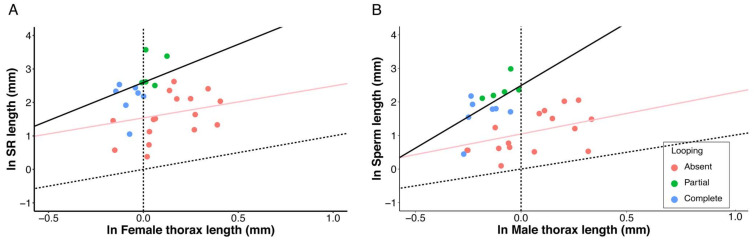
Relationships between mean (**A**) SR length and female thorax length, and (**B**) sperm length and male thorax length for all species examined. PGLS regressions of ln-transformed variables were conducted for species with partial or complete cyst looping (black lines) and for species with no cyst looping (pink lines). Slopes of these lines estimate interspecific allometric relationships. Dotted lines indicate isometry (slope = 1.0) with a y-intercept of x = 0.

Relative to outgroup species lacking cyst looping, the monophyletic lineage comprising the *saltans* and *willistoni* species groups have very long SRs and sperm for their body size (Figure 5). The evolutionary (i.e., interspecific) allometric slopes for SR length among the two groups (i.e., looping (partial and complete) or no looping) were not significantly different from one another (adjusted R^2^ = 0.11, F_3,24_ = 2.20, *p* = 0.11, λ = 0.49, looping slope = 1.32, no looping slope = 0.95), whereas species with cyst looping had a significantly higher y-intercept (*p* = 0.02; Figure 5A). These relationships for sperm length differed in that species with cyst looping had both a significantly steeper slope (adjusted R^2^ = 0.30, F_3,24_ = 4.99 *p* = 0.007, λ = 0.29, looping slope = 3.72, no looping slope = 1.02) and a significantly higher y-intercept (*p* = 0.01; Figure 5B). The interspecific allometric slope for species where looping is absent is much lower than previously reported in a study by Lüpold et. al. [21]. This disparity is likely explained by the size of the present study (28 rather than 46 species) and the absence of any of the previously examined species with gigantic sperm (e.g., *D. bifurca*, *D. kanekoi*, *D. pachea*).

**Figure 6:**
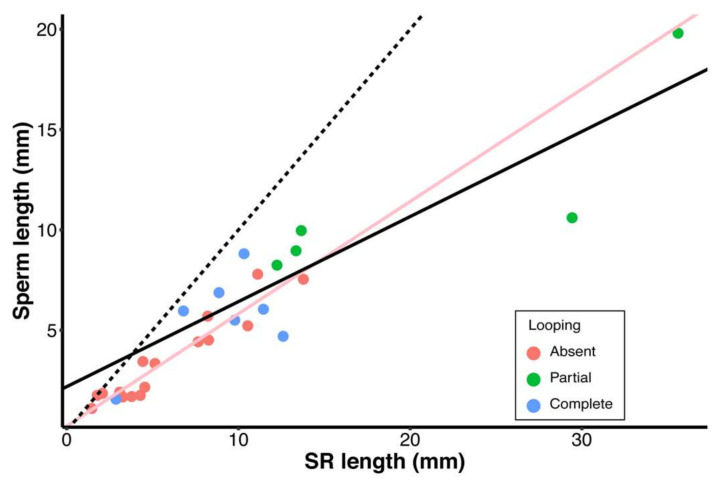
Relationship between mean sperm length and SR length for all species examined. PGLS regressions were conducted for species with partial or complete cyst looping (black line) and for species with no cyst looping (pink line). The dotted line indicates isometry (slope = 1.0) with a y-intercept of x = 0.

SR length and sperm length showed a strong positive evolutionary correlation across all species, with no influence of looping upon the relationship (adjusted R^2^ = 0.86, F_3,24_ = 55.01, *p* < 0.001, λ < 0.001; test for the effect of looping: F_1,24_ = 1.34, *p* = 0.26; Figure 6). This result corroborates previous reports showing that SR and sperm length co-diversified in a rather precise manner in *Drosophila* [20,25,35] with similar patterns observed across diverse animal taxa [12].

**Figure 7:**
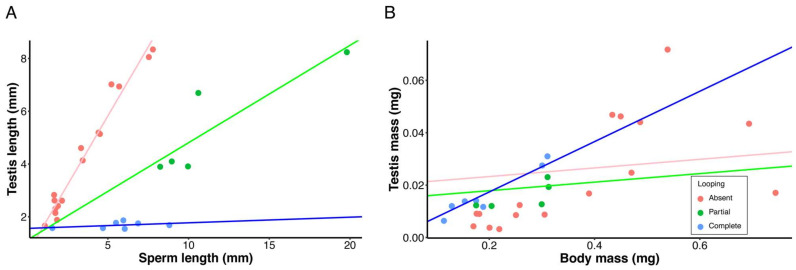
Relationships between mean (**A**) testis length and sperm length, and (**B**) dry testis mass and male dry body mass for all species examined. PGLS regressions were conducted for species with complete cyst looping (blue lines), partial cyst looping (green lines) and for species with no cyst looping (pink lines). Panel B is presented only as visual representation of the testis mass-body mass relationship, as all interpretation of the evolution of testis mass among these species is based on multiple regression (Table 2A). Note that the scatterplot represents the mean trait values for each species examined, whereas the regression lines are the lines of best fit on the PGLS regression (see Methods for details), that take into account the phylogenetic relations of the observed mean values.

When analyzing correlated evolutionary responses to cyst looping in testis investment and life-history traits, we additionally discriminated between species regarding partial versus complete looping. Perhaps the most striking result was that cyst looping decoupled the evolutionary relationship between testis length and sperm length, thereby altering what was thought to be a universal physiological constraint of spermatogenesis applicable to all *Drosophila* species [21,35]. We observed highly significant associations between sperm length, testis length and looping (adjusted R^2^ = 0.94, F_5,21_ = 90.23, *p* < 0.001, λ = 0.64). As with previous studies [21,35], we found a strong positive association between testis length and sperm length in the outgroup species with no cyst looping (slope = 1.04, *p* < 0.001; Figure 7A). This association was positive but still significant, yet significantly weaker for species with partial looping (slope = 0.37, *p* < 0.001; Figure 7A) and nearly absent in species with complete looping (slope = 0.02, *p* < 0.001; Figure 7A).

Contrary to expectation, our results revealed that species with cyst looping, despite having the relatively shortest testes (i.e., for their sperm length), did not produce sperm of a given length at a reduced energetic investment (GSI is commonly used, across diverse taxa, as an index of relative energetic investment in spermatogenesis [57]). In fact, the highest GSI for sperm of a given length tended to be made by species with complete looping (Figure 7B). Further, multiple regression revealed that a significant increase in testis mass, while controlling for body mass and sperm length, was associated with sperm looping (Table 2A, Figure 7B).

**Figure 8:**
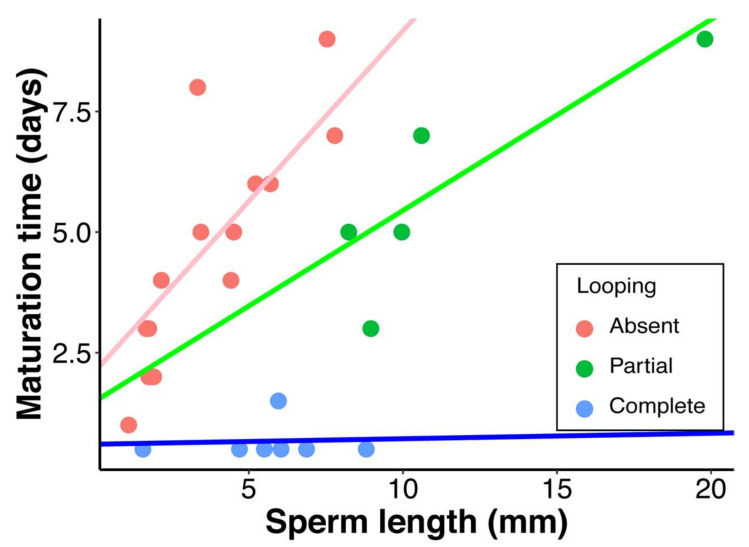
Relationships between male maturation time (days post-eclosion for sperm to occupy the seminal vesicles) and sperm length for all species examined. PGLS regressions were conducted for species with complete cyst looping (blue lines), partial cyst looping (green lines) and for species with no cyst looping (pink lines).

We found that the relationships between sperm length and two important life-history traits: (1) male time to reproductive maturity following eclosion and (2) sex-specific longevity, differed significantly between species with different patterns of sperm looping (Figure 8; Table 2B,C). Whereas species with no looping showed a strong positive relationship between maturation time and sperm length (slope = 0.44, *p* < 0.02; see Pitnick et al. 1995a), the y-intercept of this relationship was significantly lower for species with partial looping (*p* = 0.003), and the association disappeared entirely for species with complete looping (slope = 0.01). In fact, males of six out of seven species with complete looping reached reproductive maturity within 12 h of eclosion. We observed no significant association in multiple regression analysis of female median longevity over SR length between species with no looping and those with looping (Table 2B). In contrast, all species with complete looping exhibited a strong negative association between sperm length and male median lifespan (i.e., as sperm length increased, male longevity decreased; Table 2C).

## 4. Discussion

Based on our comparative immunofluorescence analysis of sperm cyst development in *D. melanogaster* and *D. willistoni*, we hypothesize that sperm cyst looping in the *willistoni* group species is a consequence of evolutionary heterochrony [58,59]. Specifically, it is induced as a result of the slower elongation rate of spermatids and cyst cells relative to the sperm tails contained within (with growth of cells and spermatid tails being more synchronized in *D. melanogaster* and other “outgroup” species). Indeed, considering that the structure of the flagellar axonemes in species that exhibit looping is indistinguishable from those that do not, sperm looping cannot be an inherent property of the sperm tails. As evident from our results, the tails within early spermatids of *D. willistoni* appeared looped because the cells have not elongated to allow full extension of the tails (Figure 3C,D). This was also the case in later stages of tail elongation, causing the tails to loop rather than becoming fully extended (Figure 3E,F). Because cellular elongation requires membrane addition [60], it is likely that flagellar axoneme assembly is favored over membrane synthesis and remodeling in species that undergo sperm looping. For this reason, it seems likely that sperm looping is induced as a by-product of delayed elongation of spermatids and overlying somatic cyst cells relative to the rate of flagellar axoneme assembly.

The observed patterns of correlated reproductive and life-history trait evolution complement one another and, in combination, they strongly support an evolutionary scenario, detailed below, for the adaptive value of sperm cyst looping. First, the *saltans* and *willistoni* group species examined here have exceptionally long SRs and sperm for their body size (Figure 5), suggesting that postcopulatory sexual selection on females and males has been intense in this lineage [21,61]. In fact, our results include the discovery that this monophyletic lineage represents an additional origin of giant sperm. *Drosophila sturtevanti* (in the *saltans* species group) has the third longest SR (35.60 mm) and fourth-longest sperm (19.80 mm) of any *Drosophila* species, and one of the longest sperm in the Kingdom Animalia (Table 1; [9,16,20]). The observed pattern of SR-sperm length co-diversification across the *saltans* and *willistoni* group species (Figure 6) importantly suggests that the selective landscape for these functionally interacting and co-diversifying traits [20,25,62] has not differed from other branches of the *Drosophila* lineage that lack cyst looping. Whereas our understanding of the adaptive value of variation in female reproductive tract traits remains highly speculative [12,21,63], the length and the extent of evolutionary diversification of SRs among the species clearly indicates intense sexual selection on sperm length in this lineage [18,21,24].

We postulate that the *willistoni* and *saltans* group species were able to evolve exceptionally long sperm for their body size as a direct energetic consequence of having derived sperm cyst looping. Consistent with previous reports, we found that all outgroup species examined had testes longer than the sperm they manufacture, resulting in a strong, positive relationship between these traits (Table 1; Figure 7A). In contrast, all of the species with looping had testes that were either equal in length to sperm (one species only, *D. tropicalis*, has very short sperm) or they were substantively shorter than the sperm they manufacture (Table 1; Figure 7A). Consequently, the testis-sperm length relationship was significantly weaker for species with partial looping and nearly absent for species with complete looping (Figure 7A). For the latter species, evolutionary increases in sperm length were accompanied by increases in the number of loops per cyst, rather than by increases in testis length (Table 1). By decoupling the relationship between testis and sperm length, the advent of cyst looping removed the allometric constraint on sperm length evolution (Figure 5A). It thus appears to be an evolutionary innovation that enables males of any species to respond to selection for longer sperm without needing to concomitantly evolve longer testes, and irrespective of their body size.

Cyst looping appears not to provide an evolutionary “free lunch,” however, since the total investment in spermatogenesis, as estimated by GSI, was significantly higher for species with complete looping (after controlling for sperm length), with no significant difference between species with partial versus no looping (Table 2A; Figure 7B). For example, the highest mean GSI, with testes representing 10.23% of total body mass, was recorded for the *willistoni* group species, *D. insularis*, which has 5.96 mm long sperm. This GSI value is the second-highest recorded in any *Drosophila* species, only exceeded by *D. bifurca*, which has 58.29 mm long sperm and a GSI of 10.60%. The increased investment in testes associated with cyst looping is not explained by the production of higher numbers of sperm, as all species with cyst looping appeared to be maturing very few sperm cysts simultaneously (S. Pitnick, personal observation), the number of sperm transferred per copulation was extremely low, and females seldom remate in nearly all of the *willistoni* and *saltans* group species (with the exception of *D. sturtevanti*; Table 1).

Given the apparent increased cost of spermatogenesis, why would selection favor cyst looping as an alternative means to responding to sexual selection for longer sperm? One answer may be that the fitness costs of looping are compensated by fitness advantages in other characters. To test this prediction, we quantified two life-history traits that are important determinants of lifetime reproductive success: age at first reproduction, and longevity. Life-history theory predicts that all else being equal, faster onset of reproduction leading to a shorter generation time will be favored by natural selection [64,65,66]. A previous comparative investigation of 42 *Drosophila* species found a significant positive relationship between sperm length and male age at reproductive maturity, which was interpreted as a constraint of the requisite energetic investment in larger, longer testes [25]. For example, the two species with the most delayed male age at first reproduction were *D. bifurca* (17 days) and *D. kanekoi* (19 days), which have 58.29 mm and 24.29 mm long sperm, respectively. Also note that age at first reproduction in females across the 42 species was unrelated to body size [25]. In striking contrast, we observed no relationship between sperm length and male maturation time among species with complete looping, with males of six out of seven species reaching sexual maturity on the day of eclosion and males of the one remaining species becoming mature the following day (Figure 8). On the other hand, there appears to be a longevity cost to complete sperm looping. Although multiple regression analysis revealed no main effect of sperm length on male longevity, there was a significant interaction between the form of cyst looping and sperm length, with male longevity significantly declining with increasing sperm length for those species with complete looping (Table 2C) [67].

Considering all the reproductive and life-history character states quantified for the 28 *Drosophila* species included in this study, we postulate that cyst looping proved selectively advantageous in the *willistoni* and *saltans* group species because it enabled the production of more competitive (i.e., longer) sperm without delaying male reproductive maturity. This benefit presumably outweighed the selective costs of looping, which include a greater energetic investment in spermatogenesis and a decline in male longevity. These results further suggest that the maturation time cost of producing longer sperm, shown to be widespread across *Drosophila* species [25,32,34], is strictly attributable to the time required to develop longer testes, rather than to the increased energetic investment.

We are not aware of any other animals with similar looping of cysts during spermatogenesis. The closest approximation has been observed in some scale insects (superfamily Coccoidea) in which the elongating flagella form a spiral encircling the central region of the spermatids [68]. A more robust understanding of the selection pressures underlying the origin and maintenance of the unique spermatogenic adaptation of cyst looping will require examination of more *willistoni* and *saltans* group species and, in particular, fieldwork on the evolutionary ecology and mating system of these species, which are distributed in the Neotropical region spanning from southern Mexico into Brazil [69,70,71]. Several of the species can be found across disparate environmental conditions ranging from savanna to tropical rain forest [72,73]. Based on the biology explored in the present study, we make two predictions regarding the evolutionary ecology of the *willistoni* and *saltans* group species [74]. First, the larval substrates will tend to limit larval development time and, hence, adult body size. This may occur when species are specialists on small and patchy or temporally ephemeral food sources (e.g., rotting mushrooms, leaves, flowers, small fruit) or, when larval resources are abundant, if population sizes are extremely large and/or interspecific competition is strong. Such conditions would similarly limit the availability of energy, stored as larval-derived fat body, to grow adult structures (i.e., testes and SRs), leading to especially protracted periods of post-eclosion sexual inactivity as requisite resources are being accrued through adult feeding (were it not for cyst looping). Second, we predict that the mating system is structured with non-overlapping generations, which can arise as a consequence of mating taking place by the new generation on the larval substrate prior to dispersal and with mate competition limited to recently-eclosed flies (rather than older immigrants). Such a mating system would place a selective premium on rapid sexual maturation while relaxing longevity selection [63,64,65], especially when females only remate after long intervals, as is generally observed for these species (Table 1).

Unfortunately, the literature contains limited information about oviposition/larval substrates for many of these species [75,76,77,78], which is critical to understanding body size selection, energy budgets, demographics, and mating systems of *Drosophila* [79,80]. Some species are fruit-breeders and known generalists (e.g., *D. paulistorum*, *D. willistoni*, *D. nebulosa*, and *D. sturtevanti*), whereas others are suspected to be specialists (e.g., *D. equinoxialis*, *D. tropicalis*, and *D. saltans*) [71] and their mating biology in nature is largely unknown.

Finally, we note that the evolution of exceptionally long SRs relative to body size in *willistoni* and *saltans* species is paradoxical, consistent with the field’s overarching lack of understanding of the forces driving female reproductive tract evolution [4,12,17,81]. SR-sperm length co-diversification in *Drosophila* may be attributable to a runaway or self-reinforcing selection process [27], driven in part by a genetic correlation between these interacting traits [21]. In this context, we wish to draw attention to the unusual structure of the SRs in the *willistoni* and *saltans* group species. Over the majority of its distal portion, the SR tubule “loops” during development, resulting in the physical integration of loops embedded within surrounding tissue (Figure 1 and Figure 4). This is different from all other *Drosophila* species examined [20]. It is pure conjecture, but it is plausible that the unique development of SRs in the *willistoni* and *saltans* group species enables the growth of relatively long SRs at lower cost, hence also contributing to the extreme sperm-SR diversification in this lineage.

## Figures and Tables

**Table 1:** Species mean for SR length, sperm length, testis length, number of loops, relative testis mass (GSI), male maturation time, remating interval, number of sperm transferred per copulation, female and male median lifespan. Colors: blue: complete looping (rows 1–7), green: partial looping (rows 8–12), pink: no looping (rows 13–28). * M = monogamous.

Species	SR Length (mm)	Sperm Length (mm)	Testis Length (mm)	Loop no.	GSI (%)	Maturity (Days)	RI_50_ (Days) *	Sperm per Copulation	Female Median Lifespan (Days)	Male Median Lifespan (Days)
*D equinoxialis*	12.60 ± 0.05	4.70 ± 0.42	1.57 ± 0.02	8	9.35	0.5	14	128 ± 6	39	32
*D nebulosa*	11.45 ± 0.27	6.05 ± 0.42	1.54 ± 0.01	7	6.20	0.5	21	118 ± 9	42	28
*D paulistorum*	10.32 ± 0.29	8.81 ± 0.53	1.68 ± 0.01	9	9.23	0.5	21	141 ± 10	42	32
*D sucinea*	9.78 ± 0.10	5.50 ± 0.29	1.77 ± 0.01	8	8.09	0.5	21	87 ± 9	35	32
*D tropicalis*	2.87 ± 0.04	1.56 ± 0.20	1.57 ± 0.01	4	5.66	0.5	21	100 ± 10	51	32
*D willistoni*	8.87 ± 0.12	6.87 ± 0.32	1.75 ± 0.02	12	9.12	0.5	14	114 ± 9	28	21
*D insularis*	6.80 ± 0.27	5.96 ± 0.39	1.86 ± 0.06	4	10.23	1.5	21	97 ± 10	42	39
*D saltans*	13.35 ± 0.17	8.96 ± 0.45	4.10 ± 0.08	7	5.83	3	21	73 ± 8	59	51
*D austrosaltans*	13.66 ± 0.22	9.96 ± 0.33	3.91 ± 0.09	9	4.26	5	7	60 ± 8	39	38
*D prosaltans*	12.24 ± 0.05	8.24 ± 0.28	3.90 ± 0.05	8	7.02	5	10	49 ± 8	51	44
*D sturtevanti*	35.60 ± 1.12	19.80 ± 0.45	8.24 ± 0.09	3	7.45	9	2	78 ± 8	36	33
*D emarginata*	29.43 ± 2.05	10.60 ± 0.72	6.69 ± 0.02	NA	6.12	7	NA	NA	NA	NA
*D americana*	10.54 ± 0.53	5.22 ± 0.02	7.02 ± 0.34	NA	6.50	6	0.5	NA	77	66
*D ananassae*	4.55 ± 0.23	2.16 ± 0.02	2.61 ± 0.11	NA	1.86	4	21	NA	43	40
*D biarmipes*	3.08 ± 0.06	1.91 ± 0.03	2.41 ± 0.05	NA	1.47	2	M	NA	42	53
*D bipectinata*	4.28 ± 0.07	1.75 ± 0.02	2.14 ± 0.07	NA	2.52	2	14	126 ± 8	36	36
*D borealis*	13.78 ± 0.17	7.54 ± 0.05	8.05 ± 0.31	NA	8.96	9	1	NA	49	45
*D immigrans*	3.78 ± 0.05	1.70 ± 0.11	2.62 ± 0.19	NA	5.28	3	M	NA	51	54
*D lummei*	11.12 ± 0.42	7.79 ± 0.02	8.34 ± 0.13	NA	6.23	7	7	NA	42	42
*D melanogaster*	2.08 ± 0.16	1.85 ± 0.01	1.91 ± 0.01	NA	5.00	2	3	1414 ± 32	45	41
*D montana*	5.14 ± 0.30	3.34 ± 0.02	4.60 ± 0.08	NA	7.87	8	1	NA	72	68
*D serrata*	4.44 ± 0.21	3.44 ± 0.09	4.14 ± 0.26	NA	5.11	5	14	NA	67	63
*D siamana*	7.65 ± 0.07	4.42 ± 0.04	5.19 ± 0.13	NA	2.31	4	21	NA	58	54
*D simulans*	1.46 ± 0.04	1.10 ± 0.01	1.66 ± 0.03	NA	4.98	1	4	1340 ± 35	27	34
*D sulfurigaster*	8.26 ± 0.08	4.51 ± 0.60	5.14 ± 0.09	NA	4.36	5	14	NA	44	47
*D suzukii*	3.27 ± 0.23	1.67 ± 0.04	2.83 ± 0.04	NA	2.90	3	7	NA	35	25
*D virilis*	8.21 ± 0.26	5.70 ± 0.16	6.94 ± 0.30	NA	8.50	6	1	NA	73	73
*D yakuba*	1.77 ± 0.03	1.75 ± 0.01	2.31 ± 0.02	NA	3.47	3	M	1068 ± 56	59	59

**Table 2:** Results of multiple regressions. * significant slopes (*p* < 0.05).

(A) Testis Mass			
Variable	β (Slope)	SE β	Partial R^2^
Body mass	0.09 *	0.03	0.60
Sperm length	0.008	0.006	0.44
Sperm length x looping			0.76
Absent	0.07	0.01	
Partial	0.09	0.02	
Complete	0.11 *	0.02	
Adjusted R-squared: 0.50; F_5,21_ = 6.09; *p* = 0.001, λ = 1^(0.93,1)^			
**(B) Female Median Lifespan**			
**Variable**	**β (slope)**	**SE β**	**Partial R^2^**
Body mass	0.53	0.42	0.009
SR length	0.01	0.03	0.02
SR length x SR looping			0.05
Absent	−0.02	0.06	
Present	−0.04	0.07	
Adjusted R-squared: 0.08; F_4,22_ = 1.61; *p* = 0.21, λ = 0^(0,0.74)^			
**(C) Male Median Lifespan**			
**Variable**	**β (slope)**	**SE β**	**Partial R^2^**
Body mass	1.82 *	0.25	0.34
Sperm length	0.07	0.06	0.07
Sperm length x looping			0.75
Absent	−0.23	0.14	
Partial	−0.29	0.20	
Complete	−0.45 *	0.22	
Adjusted R-squared: 0.34; F_5,21_ = 3.68; *p* = 0.015, λ = 0^(0,0.43)^			

## Data Availability

All data used in this study are provided as Supplementary Materials (Table S1).

## Supplementary Materials

The following are available online at https://www.mdpi.com/article/10.3390/cells10102762/s1, Figure S1: Maximum likelihood phylogeny; Table S1: Species means for all trait values not reported in text.

## References

[1] Darwin C. (1981). The Descent of Man, and Selection in Relation to Sex.

[2] Parker G.A. (2021). How soon hath time… a history of two “seminal” publications. Cells.

[3] Parker G.A. (1970). Sperm competition and its evolutionary consequences in the insects. Biol. Rev..

[4] Eberhard W.G. (1996). Female Control: Sexual Selection by Cryptic Female Choice.

[5] Parker G.A. (1979). Sexual selection and sexual conflict. Sex. Sel. Reprod. Compet. Insects.

[6] Parker G.A., Baker R.R., Smith V.G.F. (1972). The origin and evolution of gamete dimorphism and the male-female phenomenon. J. Theor. Biol..

[7] Parker G.A. (1982). Why are there so many tiny sperm? Sperm competition and the maintenance of two sexes. J. Theor. Biol..

[8] Lessells C., Snook R., Hosken D.J., Birkhead T., Hosken D., Pitnick S. (2009). The evolutionary origin and maintenance of sperm: Selection for a small, motile gamete mating type. Sperm Biology: An Evolutionary Perspective.

[9] Pitnick S., Hosken D.J., Birkhead T.R., Birkhead T., Hosken D., Pitnick S. (2009). Sperm morphological diversity. Sperm Biology: An Evolutionary Perspective.

[10] Simmons L.W. (2019). Sperm Competition and Its Evolutionary Consequences in the Insects.

[11] Fitzpatrick J.L., Lüpold S. (2014). Sexual selection and the evolution of sperm quality. Mol. Hum. Reprod..

[12] Pitnick S., Wolfner M.F., Suarez S.S., Birkhead T., Hosken D., Pitnick S. (2009). Ejaculate–female and sperm–female interactions. Sperm Biology: An Evolutionary Perspective.

[13] Chapman T. (2008). The soup in my fly: Evolution, form, and function of seminal fluid proteins. PLoS Biol..

[14] Avila F.W., Sirot L.K., LaFlamme B.A., Rubinstein C.D., Wolfner M.F. (2011). Insect seminal fluid proteins: Identification and function. Annu. Rev. Entomol..

[15] Lüpold S., Pitnick S. (2018). Sperm form and function: What do we know about the role of sexual selection?. Reproduction.

[16] Kahrl A.F., Snook R.R., Fitzpatrick J.L. (2021). Fertilization mode drives sperm length evolution across the animal tree of life. Nat. Ecol. Evol..

[17] Higginson D.M., Miller K.B., Segraves K.A., Pitnick S. (2012). Female reproductive tract form drives the evolution of complex sperm morphology. Proc. Natl. Acad. Sci. USA.

[18] Miller G.T., Pitnick S. (2002). Sperm-female coevolution in *Drosophila*. Science.

[19] Pitnick S., Spicer G.S., Markow T.A. (1995). How long is a giant sperm?. Nature.

[20] Pitnick S., Marrow T., Spicer G.S. (1999). Evolution of multiple kinds of female sperm-storage organs in *Drosophila*. Evolution.

[21] Lüpold S., Manier M.K., Puniamoorthy N., Schoff C., Starmer W.T., Luepold S.H.B., Belote J.M., Pitnick S. (2016). How sexual selection can drive the evolution of costly sperm ornamentation. Nature.

[22] Pattarini J.M., Starmer W.T., Bjork A., Pitnick S. (2006). Mechanisms underlying the sperm quality advantage in *Drosophila melanogaster*. Evolution.

[23] Lüpold S., Manier M.K., Berben K.S., Smith K.J., Daley B.D., Buckley S.H., Belote J.M., Pitnick S. (2012). How multivariate ejaculate traits determine competitive fertilization success in *Drosophila melanogaster*. Curr. Biol..

[24] Lüpold S., Reil J.B., Manier M.K., Zeender V., Belote J.M., Pitnick S. (2020). How female × male and male × male interactions influence competitive fertilization in *Drosophila melanogaster*. Evol. Lett..

[25] Pitnick S., Markow T.A., Spicer G.S. (1995). Delayed male maturity is a cost of producing large sperm in *Drosophila*. Proc. Natl. Acad. Sci. USA.

[26] Lüpold S., Pitnick S., Berben K.S., Blengini C.S., Belote J.M., Manier M.K. (2013). Female mediation of competitive fertilization success in *Drosophila melanogaster*. Proc. Natl. Acad. Sci. USA.

[27] Fisher R.A. (1930). The Genetical Theory of Natural Selection.

[28] Kokko H., Brooks R., McNamara J.M., Houston A.I. (2002). The sexual selection continuum. Proc. R. Soc. Lond. B.

[29] Henshaw J.M., Jones A.G. (2020). Fisher’s lost model of runaway sexual selection. Evolution.

[30] Amitin E.G., Pitnick S. (2007). Influence of developmental environment on male- and female-mediated sperm precedence in *Drosophila melanogaster*. J. Evol. Biol..

[31] Miller G.T., Pitnick S. (2003). Functional significance of seminal receptacle length in *Drosophila melanogaster*. J. Evol. Biol..

[32] Pitnick S. (1993). Operational sex ratios and sperm limitation in populations of *Drosophila pachea*. Behav. Ecol. Sociobiol..

[33] Pitnick S., Markow T.A. (1994). Large-male advantages associated with costs of sperm production in *Drosophila hydei*, a species with giant sperm. Proc. Natl. Acad. Sci. USA.

[34] Pitnick S., Markow T.A. (1994). Male gametic strategies: Sperm size, testes size, and the allocation of ejaculate among successive mates by the sperm-limited fly *Drosophila pachea* and its relatives. Am. Nat..

[35] Pitnick S. (1996). Investment in testes and the cost of making long sperm in *Drosophila*. Am. Nat..

[36] Pitnick S., Miller G.T., Reagan J., Holland B. (2001). Males’ evolutionary responses to experimental removal of sexual selection. Proc. R. Soc. Lond. B.

[37] Immler S., Pitnick S., Parker G.A., Durrant K.L., Lupold S., Calhim S., Birkhead T.R. (2011). Resolving variation in the reproductive tradeoff between sperm size and number. Proc. Natl. Acad. Sci. USA.

[38] White-Cooper H., Doggett K., Ellis R.E., Birkhead T., Hosken D., Pitnick S. (2009). The evolution of spermatogenesis. Sperm Biology: An Evolutionary Perspective.

[39] Fisher H.S., Jacobs-Palmer E., Lassance J.-M., Hoekstra H.E. (2016). The genetic basis and fitness consequences of sperm midpiece size in deer mice. Nat. Commun..

[40] Gimond C., Vielle A., Silva-Soares N., Zdraljevic S., McGrath P.T., Andersen E.C., Braendle C. (2019). Natural variation and genetic determinants of *Caenorhabditis elegans* sperm size. Genetics.

[41] Hime G., Brill J.A., Fuller M. (1996). Assembly of ring canals in the male germ line from structural components of the contractile ring. J. Cell Sci..

[42] Casal J., González C., Ripoll P. (1990). Spindles and centrosomes during male meiosis in *Drosophila melanogaster*. Eur. J. Cell Biol..

[43] Schindelin J., Arganda-Carreras I., Frise E., Kaynig V., Longair M., Pietzsch T., Preibisch S., Rueden C., Saalfeld S., Schmid B. (2012). Fiji: An open-source platform for biological-image analysis. Nat. Methods.

[44] O’Grady P.M., DeSalle R. (2018). Phylogeny of the genus *Drosophila*. Genetics.

[45] Katoh K., Standley D.M. (2013). MAFFT Multiple Sequence Alignment Software Version 7: Improvements in performance and usability. Mol. Biol. Evol..

[46] Stamatakis A. (2014). RAxML Version 8: A tool for phylogenetic analysis and post-analysis of large phylogenies. Bioinformatics.

[47] Paradis E., Schliep K. (2019). Ape 5.0: An Environment for modern phylogenetics and evolutionary analyses in R. Bioinformatics.

[48] Sanderson M.J. (2002). Estimating absolute rates of molecular evolution and divergence times: A penalized likelihood approach. Mol. Biol. Evol..

[49] Paradis E. (2013). Molecular dating of phylogenies by likelihood methods: A comparison of models and a new information criterion. Mol. Phylogenet. Evol..

[50] Russo C., Takezaki N., Nei M. (1995). Molecular phylogeny and divergence times of drosophilid species. Mol. Biol. Evol..

[51] Morales-Hojas R., Vieira J. (2012). Phylogenetic patterns of geographical and ecological diversification in the subgenus *Drosophila*. PLoS ONE.

[52] R Core Team R: A Language and Environment for Statistical Computing. https://www.yumpu.com/en/document/view/6853895/r-a-language-and-environment-for-statistical-computing.

[53] Orme D., Freckleton R., Thomas G., Petzoldt T., Fritz S., Isaac N., Pearse W. (2013). The Caper package: Comparative analysis of phylogenetics and evolution in R. R Package Version.

[54] Russo C.A., Mello B., Frazão A., Voloch C.M. (2013). Phylogenetic analysis and a time tree for a large drosophilid data set (Diptera: Drosophilidae). Zool J. Linnean Soc..

[55] Markow T.A., O’Grady P.M. (2006). Drosophila: A Guide to Species Identification and Use.

[56] Nonidez J.F. (1920). The internal phenomena of reproduction in *Drosophila*. Biol. Bull..

[57] Parker G.A., Pizzari T. (2010). Sperm competition and ejaculate economics. Biol. Rev..

[58] Klingenberg C.P. (1998). Heterochrony and allometry: The analysis of evolutionary change in ontogeny. Biol. Rev. Camb. Philos. Soc..

[59] Smith K.K. (2003). Time’s arrow: Heterochrony and the evolution of development. Int. J. Dev. Biol..

[60] Noguchi T., Koizumi M., Hayashi S. (2011). Sustained elongation of sperm tail promoted by local remodeling of giant mitochondria in *Drosophila*. Curr. Biol..

[61] Bjork A., Pitnick S. (2006). Intensity of sexual selection along the anisogamy–isogamy continuum. Nature.

[62] Pitnick S., Miller G.T., Schneider K., Markow T.A. (2003). Ejaculate-Female coevolution in *Drosophila mojavensis*. Proc. R. Soc. Lond. B.

[63] Orr T.J., Brennan P.L.R. (2015). Sperm storage: Distinguishing selective processes and evaluating criteria. Trends. Ecol. Evol..

[64] Roff D.A. (1992). The Evolution of Life Histories: Theory and Analysis.

[65] Stearns S.C. (1992). The Evolution of Life Histories.

[66] Charlesworth B. (1994). Evolution in Age-Structured Populations.

[67] Lemaître J.-F., Ronget V., Tidière M., Allainé D., Berger V., Cohas A., Colchero F., Conde D.A., Garratt M., Liker A. (2020). Sex differences in adult lifespan and aging rates of mortality across wild mammals. Proc. Natl. Acad. Sci. USA.

[68] Paoli F., Roversi P.F., Mercati D., Marziali L., Cocco A., Dallai R. (2015). The ultrastructure of spermiogenesis in four species of *Coccoidea* (Insecta, Homoptera). Zool. Anz..

[69] Schumacher J., Hooton D. (2010). Population genetics of two neotropical *Drosophila saltans* group species. Drosoph. Info. Serv..

[70] Bicudo H. (1978). Reproductive isolation in *Drosophila prosaltans* (*Saltans* Group). Braz. J. Genet..

[71] O’Grady P.M., Clark J.B., Kidwell M.G. (1998). Phylogeny of the *Drosophila saltans* species group based on combined analysis of nuclear and mitochondrial DNA sequences. Mol. Biol. Evol..

[72] Döge J.S., Gottschalk M.S., De Toni D.C., Bizzo L., Oliveira S.C., Valente V.L., Hofmann P.R. (2004). New records of six species of subgenus *Sophophora* (*Drosophila*, Drosophilidae) collected in Brazil. Zootaxa.

[73] Tidon R. (2006). Relationships between drosophilids (Diptera, Drosophilidae) and the environment in two contrasting tropical vegetations. Biol. J. Linn. Soc. Lond..

[74] Emlen S., Oring L. (1977). Ecology, sexual selection, and the evolution of mating systems. Science.

[75] Valadão H., Proença C.E.B., Kuhlmann M.P., Harris S.A., Tidon R. (2019). Fruit-breeding drosophilids (Diptera) in the Neotropics: Playing the field and specialising in generalism?. Ecol. Entomol..

[76] Da Mata R.A., Valadão H., Tidon R. (2015). Spatial and temporal dynamics of drosophilid larval assemblages associated to fruits. Rev. Bras. Entomol..

[77] Roque F., Hay J.D.V., Tidon R. (2009). Breeding sites of drosophilids (Diptera) in the Brazilian Savanna. I. Fallen Fruits of *Emmotum nitens* (Icacinaceae), *Hancornia speciosa* (Apocynaceae) and *Anacardium humile* (Anacardiaceae). Rev. Bras. Entomol..

[78] Pipkin S.B. (1965). The influence of adult and larval food habits on population size of Neotropical ground-feeding *Drosophila*. Am. Midl. Nat..

[79] Patterson J.T., Stone W.S. (1954). Evolution in the Genus Drosophila.

[80] Markow T.A., O’Grady P.M. (2005). Evolutionary genetics of reproductive behavior in *Drosophila*: Connecting the dots. Annu. Rev. Genet..

[81] Keller L., Reeve H. (1995). Why do females mate with multiple males? The Sexually Selected Sperm Hypothesis. Advances in The Study of Behavior.

