# Supplementary material for: Sperm Cyst “Looping”: A Developmental Novelty Enabling Extreme Male Ornament Evolution
Source: Cells. 2021 Oct 15;10(10):2762. doi: 10.3390/cells10102762 (PMC8534658; doi:10.3390/cells10102762)
Supplement: Supplementary file 1 [file cells-10-02762-s001.zip › 1378835-sp-1012/Figure S1.pdf]

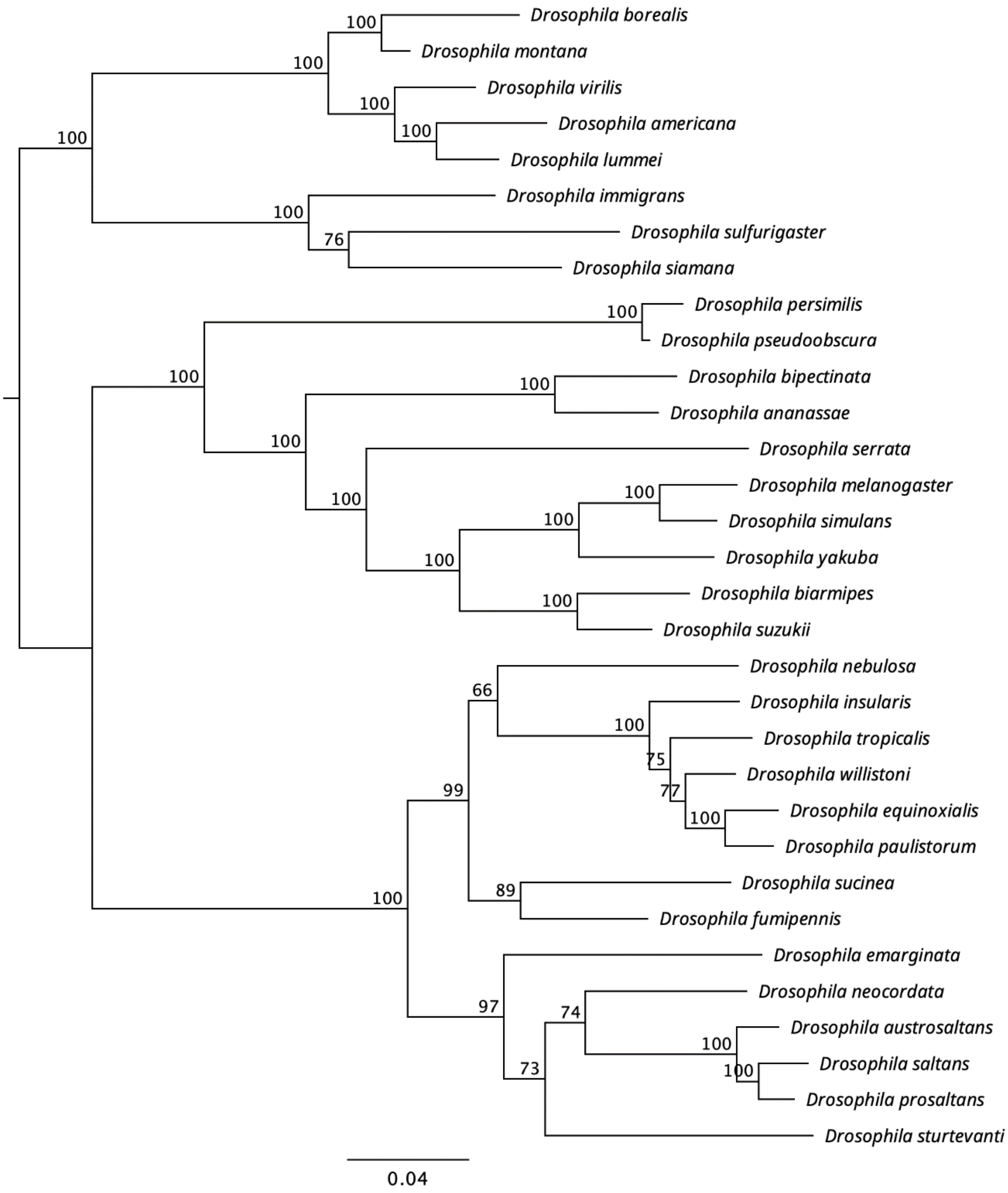

Figure S1: - Maximum likelihood (RAxML) phylogeny of our sampled taxa including a total of 32 species. Bootstrap support appears next to each node. The branch lengths indicates nucleotide substitutions per site – that is the number of changes or 'substitutions' divided by the length of the sequence.
